# Multi-Task iSpikformer for LEO Maneuver Detection and Parameter Estimation

**DOI:** 10.3390/s26206389

**Published:** 2026-10-09

**Authors:** Guo Shi, Zhongmin Pei

**Affiliations:** 1 School of Space Information, Space Engineering University, Beijing 101416, China; 2 National Key Laboratory of Space Target Awareness, Beijing 101416, China

**Keywords:** multi-task learning, spiking neural network, LEO maneuver detection

## Abstract

Low Earth orbit (LEO) maneuver analysis remains challenging because maneuver events are sparse and short in duration, while accurate analysis requires simultaneous maneuver detection, onset-time localization, and continuous three-dimensional velocity-increment (Δv) estimation from long multivariate tracking sequences. To address these challenges, an improved multi-task iSpikformer framework is proposed for the joint detection, onset-time localization, and three-dimensional velocity-increment estimation of low Earth orbit maneuvers from multivariate tracking time series. The model combines a local convolutional encoder, stacked spiking Transformer blocks, and task-specific output heads within a unified multi-task framework. Event-centered supervision, hard-negative optimization, and robust Δv regression are employed to improve sparse maneuver detection and continuous parameter estimation. For full-scene inference, predictions from overlapping windows are fused and converted into discrete maneuver events through boundary-aware event extraction and validation-based calibration. Unlike approaches that separately handle maneuver detection and parameter estimation or rely on conventional dense sequence modeling, the proposed framework jointly learns maneuver occurrence, onset location, and three-dimensional Δv from a shared temporal representation. Evaluation on a synthetic dataset of Starlink-like LEO trajectories showed that the proposed method achieved an F1-score of 0.9018, an onset-time MAE of 14.54 s, a component-wise Δv MAE of 0.0307 m/s, and a vector RMSE of 0.1302 m/s. Compared with representative baseline methods, the proposed method showed improved maneuver-detection performance and more accurate Δv estimation while maintaining comparable onset-localization accuracy. The inference-stride analysis further showed that inference time could be substantially reduced over a moderate stride range with limited changes in detection and estimation performance, whereas an excessively large stride reduced detection sensitivity. The proposed framework therefore provides a unified data-driven approach to event-level LEO maneuver analysis and demonstrates the applicability of spiking temporal modeling to joint maneuver detection and continuous orbital-parameter estimation.

## 1. Introduction

Timely detection and characterization of spacecraft maneuvers are important components of space situational awareness and orbital catalog maintenance. This requirement is particularly relevant in low Earth orbit (LEO), where the increasing number of active and maneuver-capable satellites places greater demands on tracking, orbit prediction, and data-association systems. Undetected maneuvers may cause deviations between propagated and actual trajectories, thereby affecting subsequent orbit determination and object tracking [1].

Traditional maneuver-detection methods generally rely on orbital-element historical variation, orbit-determination residuals, or sequential filtering [2,3,4,5]. Although these methods are physically interpretable, their performance may depend on observation cadence, measurement quality, dynamical-model accuracy, and the availability of post-maneuver observations. Recent data-driven studies have applied clustering, supervised learning, segmentation models, and recurrent neural networks to identify maneuver-related patterns in orbital time series [6,7,8]. The limitations of existing studies can be broadly summarized in two aspects. First, traditional model-driven methods are sensitive to observation conditions and dynamical-model accuracy. Second, many data-driven approaches focus primarily on maneuver classification or interval detection, while joint estimation of maneuver occurrence, onset time, and velocity increment remains comparatively less explored. In addition, sparse events, class imbalance, and window-boundary effects can further affect event-level detection and parameter estimation.

Transformer-based models provide an effective framework for modeling long-range dependencies in multivariate time series. The iTransformer architecture models interactions among time-series variables through an inverted token representation [9], while iSpikformer extends this structure by incorporating spiking neural network components for multivariate forecasting [10]. Nevertheless, the original iSpikformer was designed for continuous forecasting and does not directly support event-centered supervision, full-sequence event extraction, or multi-task estimation of maneuver onset and Δv.

To address these limitations, this study develops an improved multi-task iSpikformer framework for maneuver detection, onset-time localization, and Δv regression from raw LEO tracking time series. The proposed framework combines multi-task learning, event-aware supervision, and full-scene inference to jointly address maneuver detection and parameter estimation within a unified pipeline.

The main contributions of this study are summarized as follows:

- A task-oriented multi-task iSpikformer framework is developed for LEO maneuver analysis, enabling the joint learning of maneuver detection, onset-time localization, and three-dimensional Δv estimation from a shared representation of multivariate LEO tracking time series.
- An integrated task-specific optimization and full-scene inference framework is established by incorporating event-aware supervision, robust multi-task optimization, and overlap-based inference strategies. These strategies address the specific challenges of LEO maneuver estimation, including sparse maneuver events, severe sample imbalance, extreme Δv labels, and discontinuities between overlapping inference windows.
- A validation-based calibration procedure is introduced to jointly determine the detection threshold, peak prominence, minimum event distance, and boundary-exclusion margin used for boundary-aware local-peak extraction and event-level maneuver generation.

The remainder of this paper is organized as follows. Section 2 reviews related work on LEO maneuver detection, time-series event detection, and spiking neural networks. Section 3 presents the proposed multi-task iSpikformer framework. Section 4 reports the experimental results and analysis. Section 5 concludes the paper and discusses limitations and future work.

## 2. Related Work

### 2.1. LEO Maneuver Detection

Existing spacecraft maneuver-detection methods can generally be divided into orbital-history analysis, model-based estimation, and data-driven approaches. Orbital-history methods identify discontinuities or statistically unusual variations in orbital elements. Patera proposed a statistical event-detection method based on changes in historical orbital data [11]. Lemmens and Krag subsequently developed two TLE-based methods for detecting maneuvers of satellites in LEO [3]. Mukundan and Wang compared orbital elements derived from TLE data with those propagated using the SGP4 model, regarding sufficiently large deviations as potential maneuver indicators [12]. These methods can be implemented using publicly available orbital data, but their temporal resolution and reliability depend on the accuracy, update frequency, and consistency of the TLE records. Model-based approaches detect maneuvers through orbit determination, filtering, or observation association. Kelecy and Jah evaluated batch least-squares and extended Kalman filtering strategies using tracking observations from a maneuvering LEO satellite [2]. Holzinger et al. introduced control-distance metrics for object correlation, maneuver detection, and maneuver characterization under uncertain boundary conditions [13]. Other studies estimated maneuver parameters using optical survey observations [4] or radar survey observations [5]. These methods preserve a direct relationship with orbital dynamics and can provide physically interpretable estimates. However, their effectiveness depends on the assumed dynamical and measurement models, maneuver observability, tracking cadence, and the availability of observations around the maneuver epoch. Data-driven methods have also been explored to reduce reliance on manually designed detection rules. Bai et al. applied clustering algorithms to features extracted from TLE histories to identify maneuvers of different magnitudes [6]. Cipollone et al. formulated maneuver detection as a supervised temporal-segmentation problem [7]. LSTM-based models have further been used to exploit recurrent patterns in orbital and maneuver histories [14] and to estimate the probability of maneuver occurrence within a specified time horizon [8]. Although these methods provide greater flexibility in learning temporal patterns, their performance depends on the representativeness of the training data and the quality of maneuver annotations. Most existing learning-based approaches focus on classification, temporal segmentation, or maneuver-probability prediction, with comparatively limited capability for jointly determining maneuver timing and the associated Δv. In operational applications, incomplete or inaccurate maneuver characterization can propagate into orbit prediction, object correlation, tracking updates, and catalog maintenance, thereby increasing trajectory and association uncertainties.

### 2.2. Deep Learning for Time-Series Event Detection

Deep learning methods have been applied to event and anomaly detection in multivariate time series. Recurrent neural networks, particularly LSTM networks, model temporal dependencies through recurrent hidden states. Hundman et al. used LSTM prediction errors and nonparametric dynamic thresholds to detect anomalies in spacecraft telemetry from the SMAP satellite and the Mars Science Laboratory rover [15]. In orbital applications, recurrent models have also been used to learn temporal patterns associated with spacecraft maneuvers [8,14]. However, recurrent processing is inherently sequential, which can limit computational parallelism when long sequences are analyzed. Convolutional architectures provide an alternative by extracting temporal patterns through local or dilated convolutional kernels. Temporal convolutional networks have demonstrated that convolution-based models can represent long sequence dependencies and provide an effective alternative to recurrent architectures [16]. Nevertheless, their effective temporal range depends on kernel size, dilation, and network depth, and these settings may need to be adjusted when event signatures occur over different time scales. Transformer models use self-attention to model relationships between distant sequence elements without recurrent state propagation [17]. This mechanism has been adopted in forecasting, classification, and anomaly-detection tasks. Anomaly Transformer, for example, introduced an association-based mechanism for unsupervised time-series anomaly detection [18]. For multivariate forecasting, iTransformer represents each variable as a token and uses attention to learn cross-variable relationships [9]. Despite these developments, sparse-event analysis continues to face class imbalance, limited temporal localization from window-level labels, duplicated predictions in overlapping windows, and discontinuities near window boundaries. These limitations may result in missed or duplicated maneuver events and inaccurate timing or parameter estimates, which can compromise the timeliness and reliability of subsequent orbit determination, tracking updates, and maneuver assessment in operational space systems.

### 2.3. Spiking Neural Networks for Event-Driven Processing

Spiking neural networks represent information using discrete spikes and neuronal-state dynamics [19]. Their event-driven operation can reduce computational activity when spike representations are sparse, and the model is deployed on compatible neuromorphic hardware. However, practical computational and energy efficiency depends on the network architecture, spike rate, simulation procedure, and hardware platform, and therefore should be evaluated for each implementation. The non-differentiability of spike generation makes the direct optimization of deep SNNs challenging. Surrogate-gradient learning addresses this limitation by replacing the derivative of the spike function with a differentiable approximation during backpropagation [20]. This training strategy has supported the development of deeper spiking convolutional, recurrent, and attention-based models. Recent studies have combined spiking neurons with Transformer architectures. Spikformer introduced spiking self-attention and was evaluated on static and neuromorphic image-classification datasets [21]. Lv et al. subsequently developed a framework for multivariate time-series forecasting using spiking variants of recurrent, convolutional, and Transformer-based architectures [10]. Within this framework, iSpikformer was derived from iTransformer by incorporating spike encoding, spiking neurons, and softmax-free spiking self-attention. Its evaluation focused on continuous forecasting tasks involving traffic, solar-energy, and electricity-consumption data. Overall, existing spiking Transformer research has primarily addressed image classification and continuous forecasting, while its applicability to sparse maneuver events requiring both precise temporal localization and continuous physical-parameter estimation remains insufficiently validated. This uncertainty, together with the dependence of practical efficiency on spike activity and hardware implementation, may constrain the direct use of such models in space systems with stringent requirements for accuracy, latency, and computational efficiency.

## 3. Proposed Method

### 3.1. Overview

**Figure 1:**
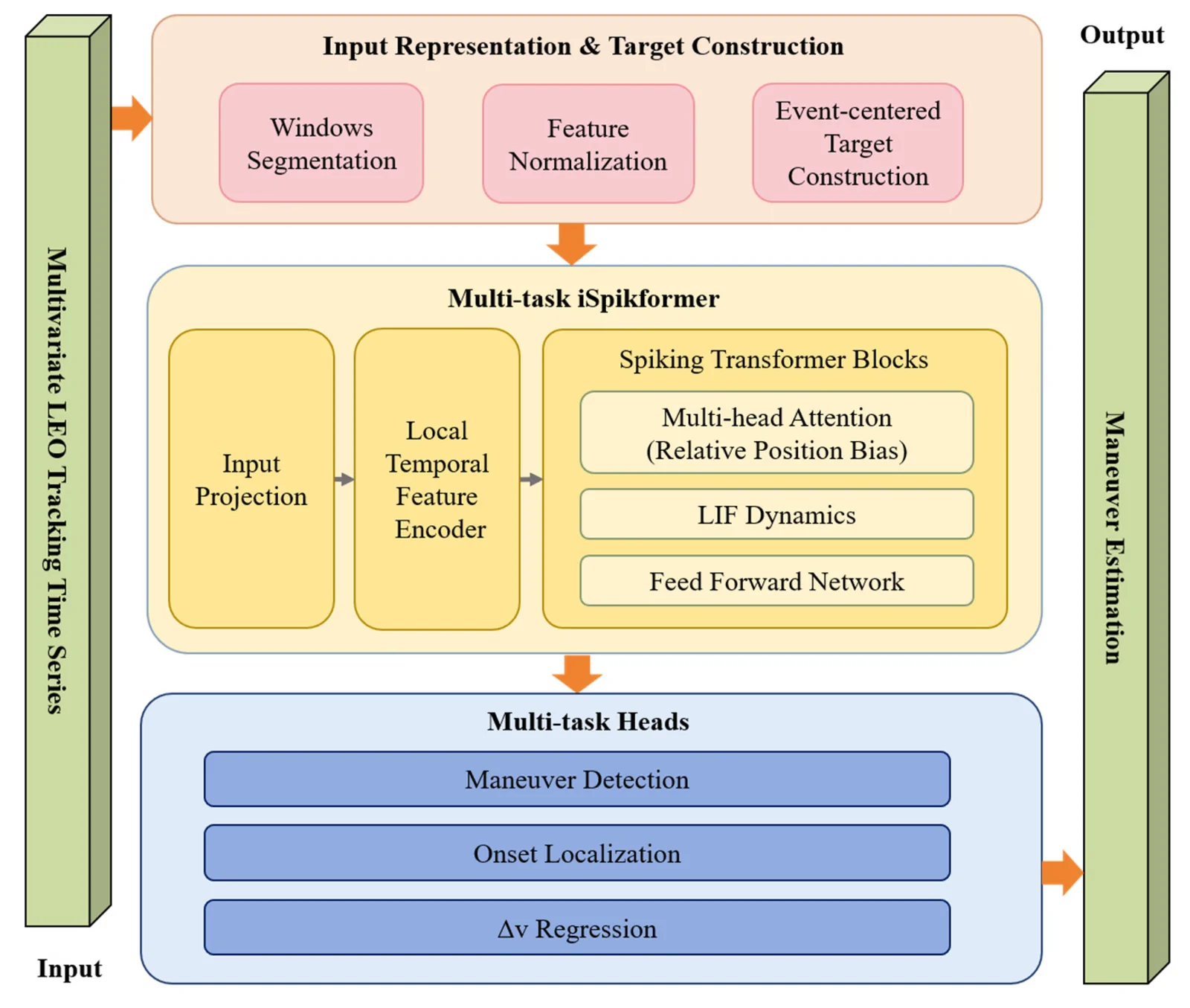
Architecture of the proposed multi-task iSpikformer.

The proposed framework is designed to jointly perform maneuver detection, onset-time localization, and Δv estimation from multivariate LEO tracking time series. As shown in Figure 1, the framework consists of three stages: input representation and target construction, multi-task spiking feature learning, and maneuver information estimation. The input sequence is first transformed into model-ready representations through window segmentation, feature normalization, and event-centered target construction. These steps enable effective learning from long tracking sequences with sparse maneuver events. The processed sequence is then fed into a multi-task iSpikformer architecture, which consists of an input projection module, a local temporal feature encoder, stacked spiking Transformer blocks, and task-specific output heads. Within the spiking Transformer blocks, multi-head attention with relative position bias, LIF neuron dynamics, and feed-forward networks are employed to model temporal dependencies in the input sequence. The shared temporal representation is subsequently mapped to three task-specific heads for maneuver detection, onset localization, and Δv regression. The outputs provide complete maneuver information, including occurrence, onset time, and velocity increment, which can support subsequent orbit analysis and space object tracking applications.

### 3.2. Problem Formulation and Event-Centered Targets

A LEO tracking scene is represented as a multivariate time series

$$X=[x_{1},\ldots ,x_{N}{]}^{T}\in R^{N\times C}$$

where N is the number of samples and C is the number of input variables. Each maneuver event e is described by an integer event center $c_{e}$, a fractional onset offset $\delta_{e}$, and a three-dimensional velocity increment $d_{e}\in R^{3}$. The continuous onset position is

$$\xi_{e}=c_{e}+\delta_{e}$$

Because one maneuver may correspond to several consecutive positive samples in the original pulse mask, each connected positive region is converted into one event. The event center is selected from the pulse region, while the corresponding offset and Δv labels are aggregated from the valid annotations within the same region.

A Gaussian-shaped target is constructed around each event center to provide localized detection supervision:

$$y_{n}=\underset{e}{max}\left[I(|n-c_{e}|\leq r)exp\left(-\frac{{\left(n-c_{e}\right)}^{2}}{2\sigma_{y}^{2}}\right)\right]$$

where r and $\sigma_{y}$ determine the target radius and temporal spread. The soft target $y_{n}$ is used for maneuver detection, whereas onset-offset and Δv regression are supervised only at the exact event centers.

For each sample, the model predicts

$$({\hat{p}}_{n},{\hat{\delta }}_{n},{\hat{d}}_{n}^{\prime })=f_{\theta }(X{)}_{n}$$

where ${\hat{p}}_{n}$ is the maneuver probability, ${\hat{\delta }}_{n}$ is the fractional onset offset, and ${\hat{d}}_{n}^{\prime }$ is the normalized Δv vector. Input variables are standardized using training-set statistics, while the three Δv components are normalized separately using robust scales derived from the training labels.

### 3.3. Multi-Task Spiking Transformer Architecture

The proposed model is an iSpikformer-inspired multi-task spiking Transformer for sample-wise maneuver analysis. The original iSpikformer was developed for multivariate time-series forecasting using spiking neural networks [10]. In the present architecture, temporal samples are processed through a local convolutional encoder and stacked spiking Transformer blocks, followed by task-specific heads for maneuver detection, onset-time localization, and Δv regression. The attention and output formulations are adapted to the requirements of maneuver-event analysis and therefore differ from those of the original forecasting architecture.

#### 3.3.1. Input and Local Feature Encoding

For an input window $X_{w}\in R^{L\times C}$, where L denotes the window length and C denotes the number of input variables, a linear projection first maps the input variables into a $d_{m}$-dimensional hidden space:

$$Z=Linear(X_{w})$$

where $Z\in R^{L\times d_{m}}$ is the projected feature representation and $d_{m}$ is the model dimension.

A depthwise-separable one-dimensional convolution is subsequently applied along the temporal dimension to capture local variations within the input window. The projected representation and the locally encoded representation are combined through a residual connection, followed by layer normalization:

$$H^{\left(0\right)}=LN\left[Z+{Conv}_{local}(Z)\right]$$

where ${Conv}_{local}(\cdot )$ denotes the local convolutional module and $H^{\left(0\right)}\in R^{L\times d_{m}}$ is the fused feature representation. The local convolutional module consists of a depthwise convolution, a pointwise convolution, and a GELU activation. The resulting representation is then replicated over $T_{s}$ internal spiking steps to form the input to the stacked spiking Transformer blocks, where $T_{s}$ denotes the number of internal spiking steps.

#### 3.3.2. Spiking Transformer Block

Each spiking Transformer block comprises pre-normalized multi-head self-attention, learnable relative-position bias, LIF neurons, residual connections, and a feed-forward network. For the h-th attention head, the attention weights are computed as

$$A_{h}=Softmax\left(\frac{Q_{h}K_{h}^{T}}{\sqrt{d_{h}}}+B_{h}^{rel}\right)$$

where $Q_{h}$, $K_{h}$, and $V_{h}$ denote the query, key, and value representations of the h-th attention head, respectively; $d_{h}$ is the feature dimension of each head; and $B_{h}^{rel}\in R^{L\times L}$ is the learnable relative-position bias. The matrix $A_{h}\in R^{L\times L}$ describes the pairwise attention weights between temporal positions within the input window.

For the q-th internal spiking step, the LIF neuron first updates its pre-reset membrane potential as

$${\tilde{u}}_{q}=u_{q-1}+\frac{a_{q}-u_{q-1}}{\tau }$$

where $a_{q}$ is the input activation and τ is the membrane time constant. A spike is generated when the membrane potential exceeds the threshold ϑ:

$$s_{q}=H\left({\tilde{u}}_{q}-\vartheta \right)$$

After spike generation, the membrane potential is reset according to

$$u_{q}={\tilde{u}}_{q}\left[1-sg(s_{q})\right]$$

where sg(⋅) denotes the stop-gradient operation. Because the Heaviside function H(⋅) is nondifferentiable, a surrogate derivative is used during backpropagation:

$$\frac{\partial s_{q}}{\partial {\tilde{u}}_{q}}\approx \frac{1}{{\left[1+\alpha |{\tilde{u}}_{q}-\vartheta |\right]}^{2}}$$

where α controls the width of the surrogate-gradient region.

The outputs of all attention heads are concatenated as

$$O={Concat}_{h}\left(A_{h}V_{h}\right)$$

where O denotes the aggregated multi-head attention representation. The attention branch and the feed-forward branch are then updated through residual connections:

$$S^{\prime }=S+W_{o}LIF(O)$$

$$S_{out}=S^{\prime }+LIF\left[FFN(LN(S^{\prime }))\right]$$

where S is the input representation of the current block, $W_{o}$ is the attention output-projection matrix, $S^{\prime }$ is the intermediate representation after the attention residual update, and $S_{out}$ is the final block output. The feed-forward network is applied independently to each temporal position.

#### 3.3.3. Multi-Task Output Heads

After the final spiking Transformer block, the hidden representations are averaged across the $T_{s}$ internal spiking steps and subsequently normalized:

$$h_{n}=LN\left(\frac{1}{T_{s}}\sum_{q=1}^{T_{s}}S_{q,n}\right)$$

where $S_{q,n}\in R^{d_{m}}$ denotes the hidden representation of the n-th temporal sample at the q-th internal spiking step, and $h_{n}\in R^{d_{m}}$ is the spike-averaged representation used by the output heads.

Three task-specific heads generate sample-wise predictions:

$${\hat{p}}_{n}=\sigma (f_{det}(h_{n}))$$

$${\hat{\delta }}_{n}=tanh(f_{off}(h_{n}))$$

$${\hat{d}}_{n}^{\prime }=f_{\Delta v}(h_{n})$$

Here, $f_{det}(\cdot )$, $f_{off}(\cdot )$, and $f_{\Delta v}(\cdot )$ denote the maneuver-detection, onset-offset, and velocity-increment heads, respectively. The scalar ${\hat{p}}_{n}\in [0,\;1]$ represents the predicted maneuver probability at sample n. The scalar ${\hat{\delta }}_{n}\in [-1,\;1]$ represents the predicted fractional onset offset relative to the integer sample position. The vector ${\hat{d}}_{n}^{\prime }\in R^{3}$ contains the three normalized components of the predicted velocity increment. The sigmoid function converts the detection logit into a probability, whereas the hyperbolic tangent function constrains the onset-offset prediction to the predefined target interval. The Δv head uses a linear output layer to produce the three normalized velocity-increment components.

Compared with previous approaches mainly designed for maneuver classification or segmentation, the proposed multi-task architecture integrates maneuver detection, onset localization, and Δv estimation within a shared representation, allowing a more comprehensive characterization of maneuver events.

### 3.4. Multi-Task Objective

The objective function combines maneuver detection, onset-offset regression, Δv regression, and false-alarm regularization:

$$L=\lambda_{det}L_{det}+\lambda_{off}L_{off}+\lambda_{\Delta v}L_{\Delta v}+\lambda_{fa}L_{fa}$$

In the implementation, the loss weights were set as $\lambda_{det}=1.0$, $\lambda_{off}=0.20$, $\lambda_{\Delta v}=0.50$, and $\lambda_{fa}=0.10$. These values were determined based on validation performance and the relative importance of different objectives. Since maneuver detection is the primary objective of the proposed framework, the detection loss was assigned the largest weight. The onset-offset and Δv regression terms provide auxiliary temporal and physical supervision, while the false-alarm regularization term uses a smaller weight to suppress spurious responses without reducing event recall. The detection term uses focal loss with hard-negative selection. Positive samples and the negative samples with the largest losses are retained, reducing the contribution of numerous easily classified nominal samples.

The two regression losses use Smooth L1 loss and are evaluated only at exact maneuver centers:

$$L_{off}=\frac{1}{|E|}\sum_{n\in E}SmoothL1({\hat{\delta }}_{n}-\delta_{n})$$

$$L_{\Delta v}=\frac{1}{|E_{v}|}\sum_{n\in E_{v}}SmoothL1({\hat{d}}_{n}^{\prime }-d_{n}^{\prime })$$

where E denotes the event-center set and $E_{v}$ contains event centers with valid Δv supervision. Extreme normalized Δv labels may be clipped or excluded from the Δv loss without removing their detection and onset supervision.

To improve training stability, the regression objectives are introduced through a warm-up strategy. During the initial training stage, the effective weights of the onset-offset and Δv regression losses gradually increase from zero to their target values, allowing the detection branch to first learn robust maneuver representations before incorporating regression supervision.

The false-alarm term is the mean predicted maneuver probability in samples whose soft targets are close to zero:

$$L_{fa}=\frac{1}{|N_{0}|}\sum_{n\in N_{0}}{\hat{p}}_{n}$$

This term regularizes the detection output in nominal regions but does not replace event-level post-processing.

The proposed objective function incorporates detection and regression supervision within a unified optimization framework, which helps address the challenges associated with sparse maneuver events, class imbalance, false alarms, and continuous parameter estimation.

### 3.5. Full-Scene Inference and Event Extraction

Complete scenes are processed using overlapping windows. To reduce window-boundary discontinuities, the outputs are combined using a hat-shaped weight that assigns larger weights to central window positions:

$${\bar{q}}_{n}=\frac{\sum_{w\in W\left(n\right)}w_{n-s_{w}}q_{n-s_{w}}^{w}}{\sum_{w\in W\left(n\right)}w_{n-s_{w}}}$$

where q denotes the maneuver probability, onset offset, or normalized Δv, and $s_{w}$ is the starting index of window w.

The aggregated probability sequence is smoothed using a Gaussian kernel. Discrete maneuver events are then obtained by local-peak extraction subject to four parameters: probability threshold, peak prominence, minimum event distance, and boundary-exclusion margin. These parameters are selected jointly on a held-out validation set.

For a detected peak at sample ${\hat{k}}_{e}$, the continuous onset estimate is

$${\hat{\xi }}_{e}=clip\left({\hat{k}}_{e}+{\bar{\delta }}_{{\hat{k}}_{e}},0,N-1\right)$$

The onset time is obtained from the scene time axis, and the physical velocity increment is recovered by reversing the component-wise normalization:

$${\hat{\Delta v}}_{e}=s_{\Delta v}⊙{\bar{d}}_{{\hat{k}}_{e}}^{\prime }$$

This procedure converts the sample-wise multi-task outputs into a discrete event list containing the detected maneuver epoch, confidence, and estimated Δv.

The full-scene inference strategy considers the effects of overlapping-window processing and reduces the influence of boundary discontinuities and duplicated predictions when generating event-level maneuver information. The complete procedure is summarized in Algorithm 1.
**Algorithm 1** Multi-Task iSpikformer for LEO Maneuver Analysis**Input**: LEO tracking scene set D; window length L; internal spiking steps $T_{s}$; epochs E; batch size B**Output**: Trained model parameters $\Theta^{*}$; calibrated event-extraction parameters $\phi^{*}$; maneuver-event predictions $\hat{y}$1: **for** each scene X∈D
**do**2:  Identify connected positive regions in the maneuver pulse mask3:  **for** each maneuver event e
**do**4:    $\left(c_{e},\delta_{e},d_{e})\leftarrow AggregateEventLabels\;(e\right)$
5:  **end for**6:  $y_{n}\leftarrow BuildGaussianTargets\;(\{c_{e}\},r,\sigma_{y})$
7:  $E\leftarrow \{c_{e}\}$,  $E_{∆v}\leftarrow \{c_{e}\in E:c_{e}\;\mathrm{has}\;\mathrm{valid}\;\Delta v\;\mathrm{supervision}\}$
8: **end for**9: Split D into $D_{train}$, $D_{val}$, and $D_{test}$
10: Standardize the input variables and normalize the Δv labels using training-set statistics and $s_{\Delta v}$
11: Initialize model parameters Θ
12: **for** epoch =1 to E
**do**13:  Generate training windows $X_{w}\in R^{L\times C}$
14:  **for** each mini-batch **do**15:    $Z\leftarrow Linear\;(X_{w})$
16:    $H^{\left(0\right)}\leftarrow LN\;\left[Z+{Conv}_{local}(Z)\right]$
17:    $S\leftarrow Repeat\;(H^{\left(0\right)},T_{s})$
18:    **for** each spiking Transformer block **do**19:      (Q,K,V)←LinearQKV [LN (S)]
20:      $A_{h}\leftarrow Softmax\left(\frac{Q_{h}K_{h}^{T}}{\sqrt{d_{h}}}+B_{h}^{rel}\right)$
21:      $O\leftarrow {Concat}_{h}(A_{h}V_{h})$
22:      $S^{\prime }\leftarrow S+W_{o}LIF\;(O)$
23:      $S\leftarrow S^{\prime }+LIF\;\{FFN\;[LN\;(S^{\prime })]\}$
24:    **end for**25:    $h_{n}\leftarrow LN\;\left(T_{s}^{-1}\sum_{q=1}^{T_{s}}S_{q,n}\right)$
26:    ${\hat{p}}_{n}\leftarrow \sigma [f_{det}(h_{n})]$,  ${\hat{\delta }}_{n}\leftarrow tanh\;[f_{off}(h_{n})]$, ${\hat{d}}_{n}^{\prime }\leftarrow f_{\Delta v}(h_{n})$
27:    $L_{det}\leftarrow HardNegativeFocalLoss\;({\hat{p}}_{n},y_{n})$
28:    $L_{off}\leftarrow |E{|}^{-1}\sum_{n\in E}SmoothL1\;({\hat{\delta }}_{n}-\delta_{n})$
29:    $L_{\Delta v}\leftarrow (3|E_{∆v}|{)}^{-1}\sum_{n\in E_{v}}\sum_{j=1}^{3}SmoothL1\;({\hat{d}}_{n,j}^{\prime }-d_{n,j}^{\prime })$
30:    $N_{0}\leftarrow \{n:y_{n}<\epsilon_{fa}\}$,  $L_{fa}\leftarrow |N_{0}{|}^{-1}\sum_{n\in N_{0}}{\hat{p}}_{n}$
31:    $L\leftarrow \lambda_{det}L_{det}+\lambda_{off}L_{off}+\lambda_{\Delta v}L_{\Delta v}+\lambda_{fa}L_{fa}$
32:    Update Θ by backpropagation with surrogate gradients33:  **end for**34:  Obtain full-scene validation outputs and calibrate ϕ∈Φ
35:  Save $\left(\Theta ,\phi \right)$ if the event-level validation performance improves36: **end for**37: $\left(\Theta^{*},\phi^{*})\leftarrow LoadBestValidationConfiguration\;(\right)$
38: **for** each test scene $X\in D_{test}$
**do**39:  $\left({\bar{p}}_{n},{\bar{\delta }}_{n},{\bar{d}}_{n}^{\prime })\leftarrow FullSceneInference\;(X,\Theta^{*}\right)$
40:  ${\tilde{p}}_{n}\leftarrow GaussianSmooth\;({\bar{p}}_{n})$
41:  $\hat{K}\leftarrow ExtractLocalPeaks\;({\tilde{p}}_{n};\phi^{*})$
42:  **for** each detected peak ${\hat{k}}_{e}\in \hat{K}\;$**do**43:    ${\hat{\xi }}_{e}\leftarrow clip\;({\hat{k}}_{e}+{\bar{\delta }}_{{\hat{k}}_{e}},0,N-1)$
44:    ${\hat{t}}_{e}\leftarrow SampleToTime\;({\hat{\xi }}_{e})$, ${\hat{\Delta v}}_{e}\leftarrow s_{\Delta v}⊙{\bar{d}}_{{\hat{k}}_{e}}^{\prime }$
45:    Add $\left({\hat{t}}_{e},{\tilde{p}}_{{\hat{k}}_{e}},{\hat{\Delta v}}_{e}\right)$ to $\hat{y}$
46:  **end for**47: **end for**48: **return**
$\Theta^{*}$, $\phi^{*}$, and $\hat{y}$


## 4. Experiments

### 4.1. Experimental Setup

#### 4.1.1. Dataset

The dataset used in this study is the Starlink-like subset of the synthetic LEO satellite trajectory dataset introduced by Baudier et al. [22], which is publicly available through Zenodo (https://doi.org/10.5281/zenodo.13885584; accessed on 15 June 2026). This dataset contains 150 synthetic satellite trajectory scenarios generated using the Orekit flight dynamics library. The trajectory simulations incorporate high-fidelity perturbation models, including non-spherical gravity, atmospheric drag, third-body gravitational effects, solar radiation pressure, and relativistic effects. Each scenario consists of a 12-h time series represented by osculating equinoctial orbital elements with a fixed sampling interval. The dataset provides maneuver annotations, including maneuver occurrence information and corresponding three-dimensional Δv vectors.

Following the original dataset protocol, the scenario-level training and testing partition is preserved, where each trajectory scenario is assigned exclusively to either the training or test set. The original training set is further divided into a training subset and a validation subset for model selection and hyperparameter tuning, while the test set remains unchanged for final evaluation. All input features are standardized using the mean and standard deviation calculated from the training subset only.

#### 4.1.2. Implementation Details

The implementation settings are summarized in Table 1. All experiments were implemented using Python (version 3.8; Python Software Foundation, Wilmington, DE, USA) in PyCharm Community Edition (version 2024.3.5; JetBrains s.r.o., Prague, Czech Republic). The input trajectories were divided into overlapping windows of 100 samples. The model was trained for up to 100 epochs using AdamW with an initial learning rate of $3\times 10^{-4}$, a weight decay of $10^{-3}$, and a cosine schedule with three warm-up epochs. Gradient clipping and exponential moving average were applied with a maximum norm of 1.0 and a decay factor of 0.999, respectively. The Δv targets were normalized using training-set statistics, with extreme labels handled according to the predefined outlier policy.

#### 4.1.3. Evaluation Metrics

The performance of the proposed framework was evaluated from three aspects: maneuver detection, onset-time localization, and  Δv estimation.

Maneuver detection: Global F1-score, scene-level macro F1-score, precision, and recall were used as evaluation metrics. A detected maneuver event was considered correct when the offset-corrected onset time was within the predefined event matching tolerance of the annotated event center.

Onset-time localization: The mean absolute error (MAE) of the estimated maneuver onset time was calculated in seconds for correctly detected events.

Δv estimation: The component-wise MAE (m/s), magnitude MAE (m/s), and angular direction error (degrees) were calculated. These metrics were evaluated only on valid events satisfying the predefined Δv outlier criterion, where extreme normalized Δv components were excluded from the regression evaluation.

### 4.2. Results

#### 4.2.1. Overall Maneuver Detection Performance

At the event level, the proposed method achieved a precision of 0.9411, a recall of 0.8656, and an F1-score of 0.9018, as shown in Table 2. Under scene-wise macro-averaging, where each test scenario contributes equally, the precision, recall, and F1-score were 0.9328, 0.8944, and 0.9071, respectively. The difference between the event-level and scene-wise macro-averaged F1-scores was 0.0053, indicating that the overall evaluation was not substantially affected by the aggregation strategy.

**Figure 2:**
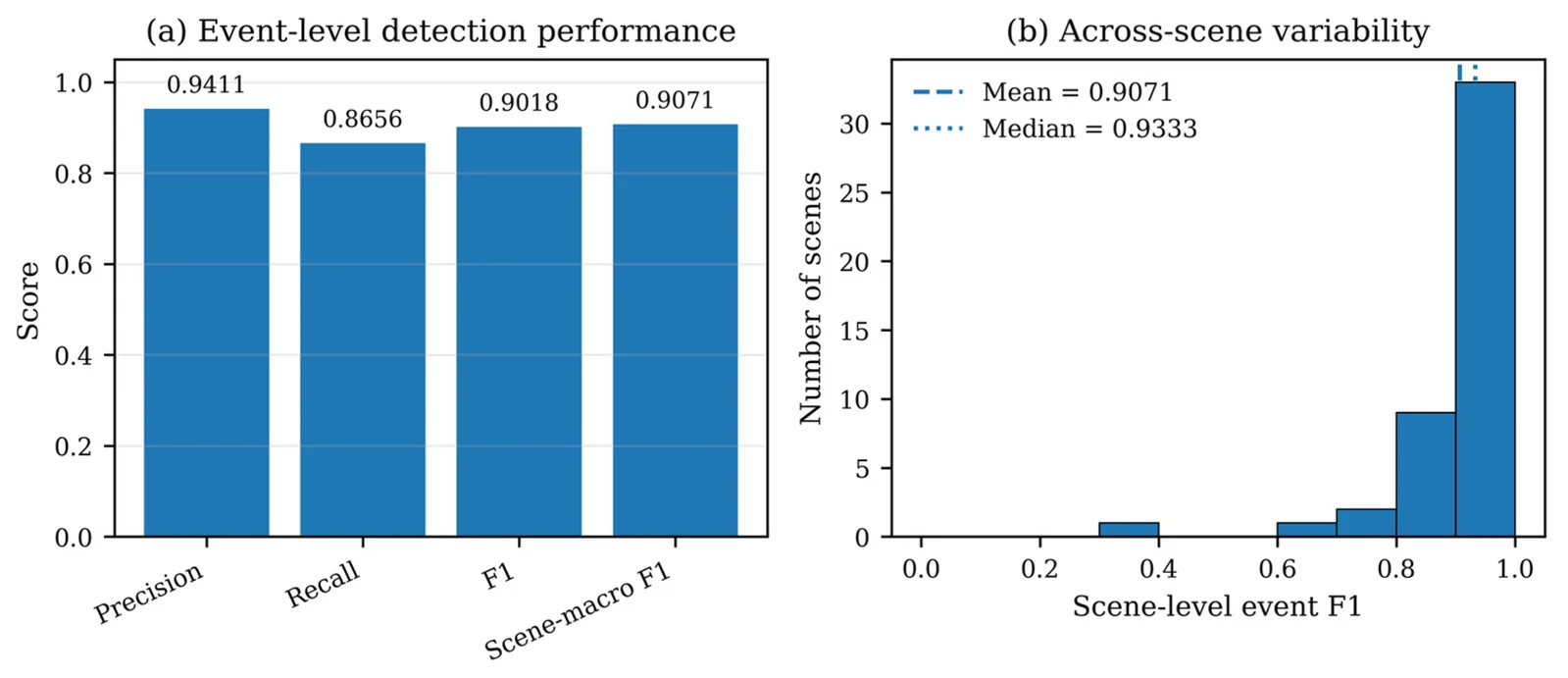
Overall detection performance and across-scenario variability on the test set.

Figure 2 presents the detection performance and variability across individual test scenarios. Most scenario-level F1-scores were distributed between 0.8 and 1.0, while several scenarios exhibited lower values, indicating performance variation among different trajectory conditions. The mean and median scenario-level F1-scores were 0.9071 and 0.9333, respectively.

#### 4.2.2. Onset-Time Localization Performance

**Figure 3:**
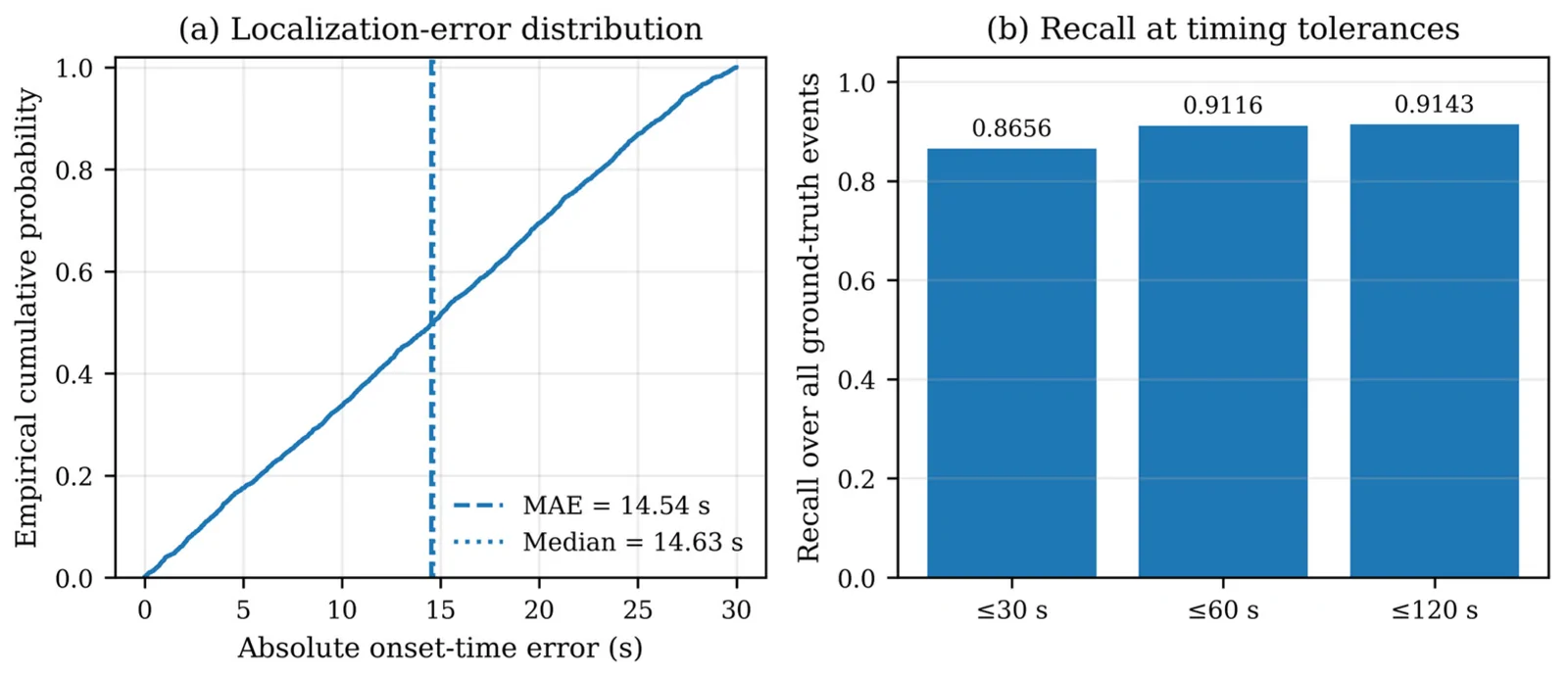
Onset-time localization performance.

The onset-time localization results are summarized in Table 3. Under the primary event-matching tolerance of 30 s, the mean absolute error and median absolute error of the matched events were 14.54 and 14.63 s, respectively. As shown in Figure 3, approximately half of the correctly matched events exhibited onset-time errors below 14.63 s. When the matching tolerance increased from 30 s to 60 s, the recall increased from 86.56% to 91.16%. Further increasing the tolerance to 120 s resulted in a recall of 91.43%, indicating that most additional matched events were obtained within the first tolerance extension.

#### 4.2.3. Δv Estimation Performance

The Δv estimation results for matched inlier events are summarized in Table 4. The proposed framework achieved a component-wise MAE of 0.0307 m/s, a vector RMSE of 0.1302 m/s, a magnitude MAE of 0.0483 m/s, and a mean angular deviation of 14.12°.

**Figure 4:**
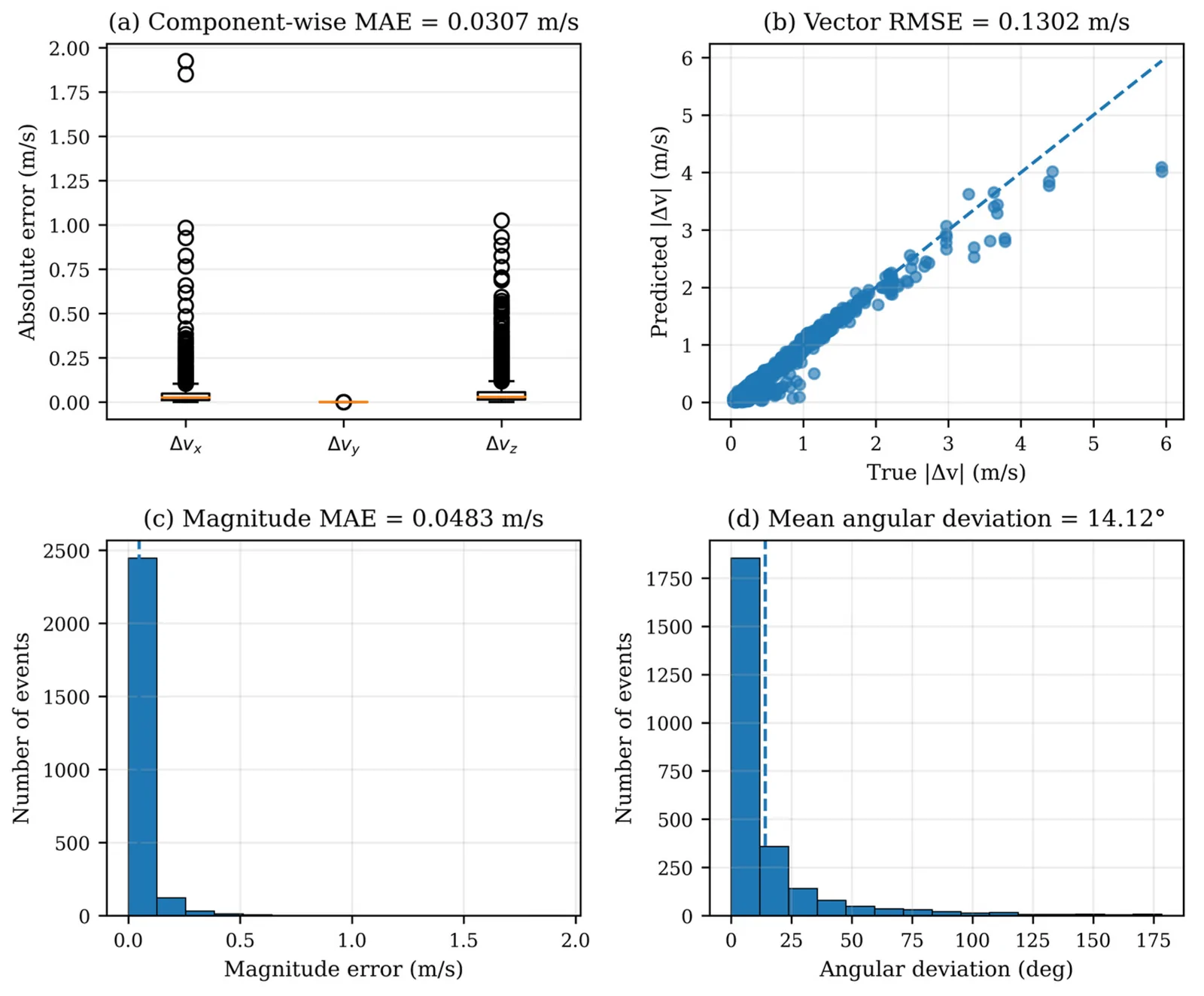
Δv estimation errors for matched inlier events.

Figure 4 presents the component-wise error distributions and the relationship between predicted and reference Δv magnitudes. Most errors were concentrated near zero, while larger deviations occurred for a limited number of events, particularly for higher-magnitude velocity increments.

#### 4.2.4. Baseline Comparison

To further evaluate the performance of the proposed framework, comparisons were conducted with representative deep learning baselines, including CNN, TCN, BiLSTM, and Transformer models. A non-spiking variant of iSpikformer was also evaluated under otherwise identical settings to isolate the contribution of the spiking mechanism. The results are summarized in Table 5. The proposed iSpikformer achieved an event-level F1-score of 0.9018, compared with 0.9069 for the non-spiking variant, while remaining higher than those of the conventional baseline models. The onset-time localization errors were comparable among different models, with the proposed iSpikformer achieving an MAE of 14.5393 s, compared with 14.6880 s for its non-spiking counterpart. For Δv estimation, the proposed framework achieved the lowest component-wise MAE (0.0307 m/s) and vector RMSE (0.1302 m/s) among the evaluated methods. Compared with the non-spiking variant, these values correspond to reductions of approximately 2.23% and 1.66%, respectively, indicating a modest improvement in Δv estimation accuracy associated with the spiking formulation. The event-level detection outcomes under the 30 s matching tolerance are further summarized in Table 6. The proposed framework identified 2635 matched events, with 165 false positives and 409 missed events, compared with 2620, 114, and 424, respectively, for the non-spiking variant. Thus, the spiking variant detected 15 additional matched events and reduced missed events by approximately 3.54%, although at the cost of more false positives. The corresponding results of the baseline models show different balances between matched events, false positives, and missed events. The average spike rate of the proposed iSpikformer was 49.61%, while the non-spiking variant and other conventional deep learning baselines did not involve spiking neuron operations and therefore did not have corresponding spike-rate measurements. The measured spike rate reflects the sparse event-driven activation characteristic of the spiking architecture. Overall, the controlled comparison indicates that the spiking mechanism improves Δv estimation accuracy and event coverage, while introducing a slight trade-off in F1-score and false positives.

#### 4.2.5. Ablation Analysis

An ablation study was conducted to evaluate the contribution of key components in the proposed framework. The results are presented in Table 7. Removing event-centered targets reduced the F1-score from 0.9018 to 0.8633. Excluding hard-negative optimization further decreased the F1-score to 0.8041. Removing the local convolutional encoder resulted in the largest performance reduction, with an F1-score of 0.7635. These results indicate that each component contributes to the final performance, with event-aware supervision, hard-negative selection, and local temporal feature extraction affecting different aspects of maneuver analysis.

#### 4.2.6. Inference-Stride Analysis

**Figure 5:**
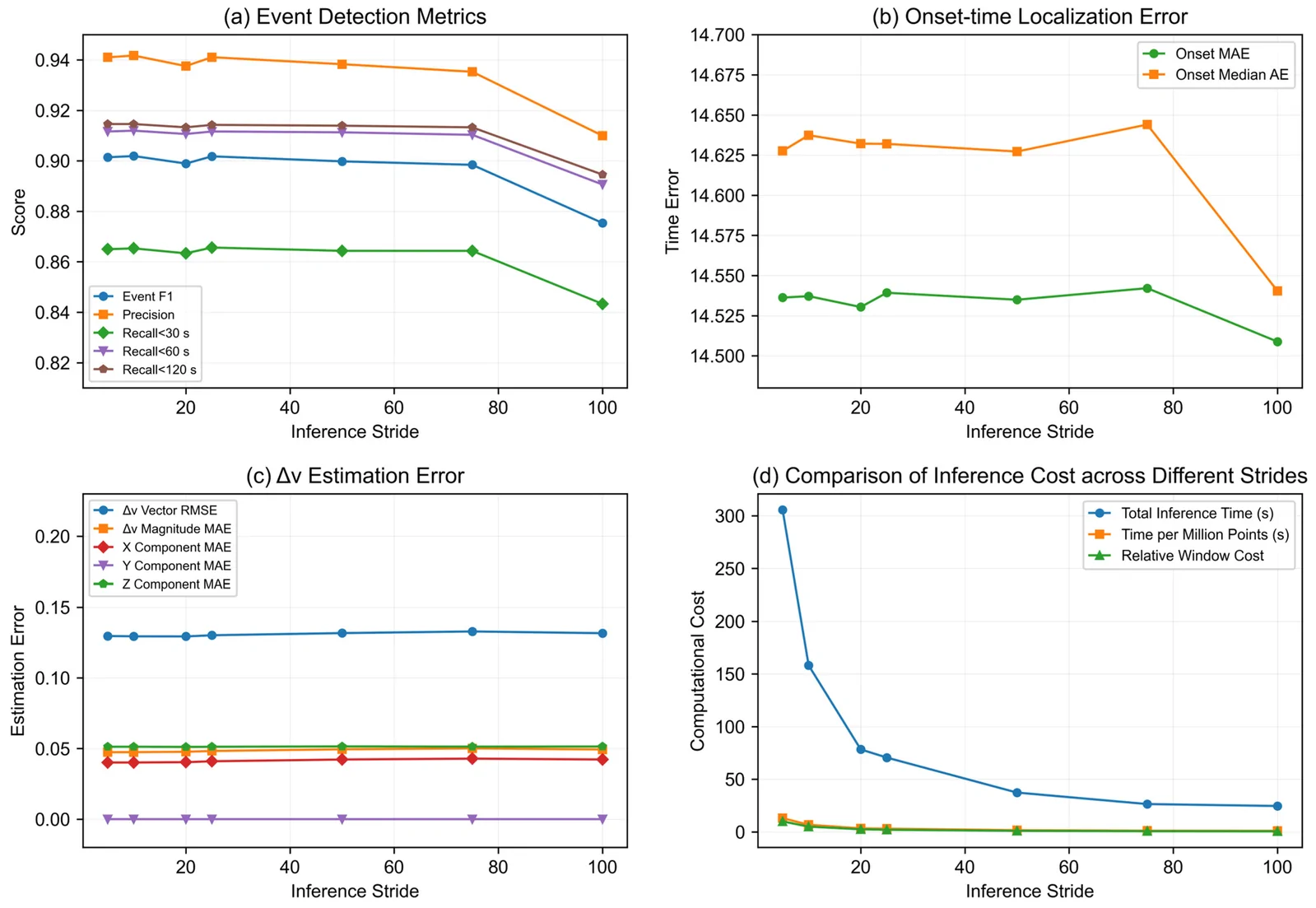
Comparison of detection performance, onset-time localization error, Δv estimation error, and computational cost across different inference strides.

The effects of inference stride on prediction performance and computational cost are summarized in Figure 5 and Table 8. For inference strides between 5 and 75 samples, the F1-score remained within 0.8984–0.9019, while onset-time and Δv estimation errors showed limited variation. Increasing the stride reduced inference time substantially. For example, increasing the stride from 5 to 25 reduced inference time from 305.53 s to 70.60 s. When the stride increased to 100 samples, the inference time was further reduced; however, the F1-score decreased to 0.8754, accompanied by reduced recall. Therefore, a stride of 25 samples was selected as the default configuration because it provided a balance between computational efficiency and prediction performance.

### 4.3. Discussion

#### 4.3.1. Strengths of the Proposed Framework

The proposed framework provides a unified approach for LEO maneuver analysis by integrating maneuver detection, onset-time localization, and Δv estimation within a single architecture. Compared with approaches that mainly focus on event detection or classification, the proposed multi-task design enables maneuver occurrence, temporal information, and physical parameters to be learned from a shared temporal representation. The comparison with baseline methods indicates that the proposed framework achieves a competitive F1-score and lower Δv estimation errors than the conventional baseline models. The controlled comparison with the non-spiking variant further shows that the spiking model achieves lower onset-time and Δv estimation errors and identifies more matched events, although with a slightly lower F1-score and more false positives. These results suggest that incorporating task-specific regression objectives into the detection framework provides additional capability for continuous maneuver-parameter estimation, while the spiking mechanism introduces a trade-off between event coverage and detection performance. The ablation analysis further shows that event-centered targets, hard-negative selection, and local temporal encoding contribute to the overall performance. These components are designed to address specific characteristics of maneuver analysis, including sparse event occurrence, imbalance between maneuver and nominal samples, and local temporal variations in tracking sequences. In addition, the proposed spiking representation exhibits sparse activation characteristics, with an average spike rate of 49.61%. This observation indicates the potential of spiking computation for reducing redundant activations.

#### 4.3.2. Limitations

Several limitations should be considered in the current study. First, the proposed framework was evaluated using the Starlink-like subset of a synthetic LEO trajectory dataset. Although the dataset incorporates high-fidelity orbital perturbations and provides controlled maneuver annotations, the generalization capability under real tracking observations and different measurement uncertainties requires further investigation. Second, the current evaluation mainly considers single-satellite maneuver analysis based on multivariate tracking time series. More complex operational scenarios, such as incomplete observations, multiple interacting objects, and highly irregular maneuver patterns, were not included in this study. Third, although the spiking architecture shows sparse activation characteristics, the practical advantages in computational efficiency have not been directly evaluated. Hardware-level assessments, including inference latency, memory consumption, and energy efficiency, are required to further characterize the applicability of the proposed framework.

## 5. Conclusions

This study proposed a multi-task spiking Transformer framework for joint maneuver detection, onset-time localization, and three-dimensional velocity-increment estimation from multivariate LEO tracking time series. The framework combines local temporal encoding, spiking Transformer blocks, task-specific prediction heads, and overlap-based full-scene inference to generate event-level maneuver estimates. On the evaluated synthetic LEO trajectory dataset, the proposed framework achieved an event-level F1-score of 0.9018, an onset-time MAE of 14.54 s, a component-wise Δ*v* MAE of 0.0307 m/s, and a vector RMSE of 0.1302 m/s. Compared with the evaluated baseline models, the proposed method achieved competitive maneuver detection performance and lower Δ*v* estimation errors. The inference-stride analysis further showed that increasing the stride from 5 to 25 reduced the inference time from 305.53 s to 70.60 s while maintaining similar detection and estimation performance. The proposed framework provides a unified approach for maneuver analysis by integrating event detection, temporal localization, and physical parameter estimation within a shared architecture. The observed average spike rate of 49.61% indicates sparse activation characteristics of the spiking representation; however, the practical efficiency advantages associated with this property require further validation.

Several limitations remain. The current findings are based on a specific dataset, annotation procedure, event-matching criterion, and validation-calibrated post-processing configuration. The current evaluation is limited to the Starlink-like subset of a publicly available synthetic dataset, in which the observation conditions, noise characteristics, and maneuver scenarios are predefined. Although the dataset incorporates high-fidelity orbital perturbations, the generalizability and robustness of the proposed framework under broader noise conditions, observation settings, and maneuver patterns have not yet been systematically evaluated. In addition, estimation errors were evaluated mainly for successfully matched events, and the computational and energy-efficiency benefits of the spiking architecture were not directly measured on dedicated hardware. Future work should evaluate the framework on more diverse datasets and systematically analyze its robustness under different observation and maneuver scenarios. Further efforts should also focus on improving the detection of difficult events, developing adaptive inference and post-processing strategies, and conducting comprehensive efficiency evaluations, including computational complexity, parameter count, hardware-level latency, memory consumption, and energy consumption, to further characterize the practical advantages of the spiking architecture.

## Figures and Tables

**Table 1:** Model Configuration.

Parameter	Value	Description
Input window length	100	Temporal window size
Hidden dimension	64	Hidden representation dimension
Feed-forward dimension	128	FFN dimension
Attention heads	4	Number of attention heads
Spike steps	2	Internal spiking steps
Batch size	32	Training batch size
Epochs	100	Maximum training epochs
Focal loss parameter	2.0	Focal loss focusing parameter
Hard negative ratio	8.0	Hard-negative selection ratio
Gaussian target sigma	0.8	Target distribution parameter
Detection threshold range	0.05–0.995	Event extraction calibration range

**Table 2:** Overall detection performance on the test set.

Evaluation Level	Precision	Recall	F1-Score
Event-level	0.9411	0.8656	0.9018
Scene-wise Macro-average	0.9328	0.8944	0.9071

**Table 3:** Onset-time localization accuracy and recall under different matching tolerances.

MAE(s)	MedAE (s)	Recall Within 30 s (%)	Recall Within 60 s (%)	Recall Within 120 s (%)
14.54	14.63	86.56	91.16	91.43

**Table 4:** Δv estimation performance for matched inlier events.

Overall Component-Wise MAE (m/s)	Vector RMSE (m/s)	Magnitude MAE (m/s)	Mean Angular Deviation (°)
0.0307	0.1302	0.0483	14.12

**Table 5:** Comparison with baseline methods.

Method	F1-Score	Onset MAE (s)	Component-Wise Δv MAE (m/s)	Vector RMSE (m/s)	Average Spike Rate (%)
iSpikformer (ours)	0.9018	14.5393	0.0307	0.1302	49.61%
iSpikformer (non-spiking)	0.9069	14.6880	0.0314	0.1324	-
CNN	0.8646	14.1362	0.1693	0.5509	-
TCN	0.8490	14.4703	0.0887	0.2710	-
BiLSTM	0.8538	13.6430	0.0773	0.2389	-
transformer	0.8463	14.4880	0.0511	0.1565	-

**Table 6:** Event-level detection outcomes under the 30 s matching tolerance.

Method	Matched Events	False Positives	Missed Events
iSpikformer (ours)	2635	165	409
iSpikformer (non-spiking)	2620	114	424
CNN	2478	210	566
TCN	2347	138	697
BiLSTM	2441	233	603
transformer	2334	138	710

**Table 7:** Ablation results.

Variant	F1-Score	Precision	Recall	Matched Events	False Positives	Missed Events	Onset MAE (s)	Component-Wise Δv MAE (m/s)	Vector RMSE (m/s)	ΔF1-Score (vs. Full Model)
Full model (ours)	0.9018	0.9411	0.8656	2635	165	409	14.5393	0.0307	0.1302	-
w/o Event Target	0.8633	0.9349	0.8019	2441	170	603	14.3569	0.0294	0.1296	−0.0385
w/o Hard Negative	0.8041	0.9211	0.7135	2172	186	872	14.1395	0.0273	0.1246	−0.0977
w/o Local Conv	0.7635	0.8645	0.6836	2081	326	963	13.4776	0.0381	0.1597	−0.1383

**Table 8:** Performance and computational efficiency under different inference strides.

Inference Stride	F1-Score	Onset MAE (s)	Recall Within 30 s (%)	Recall Within 60 s (%)	Recall Within 120 s (%)	Δv Vector RMSE (m/s)	Δv Magnitude MAE (m/s)	Angular Error (°)	Inference Time (s)
5	0.9014	14.5362	86.50	91.16	91.46	0.1296	0.0474	14.10	305.5292
10	0.9019	14.5372	86.53	91.20	91.46	0.1294	0.0475	14.11	158.1653
20	0.8989	14.5304	86.33	91.06	91.33	0.1293	0.0477	13.86	78.3645
25	0.9018	14.5393	86.56	91.16	91.43	0.1302	0.0483	14.12	70.6003
50	0.8998	14.5349	86.43	91.13	91.39	0.1317	0.0495	14.25	37.3513
75	0.8984	14.5421	86.43	91.03	91.33	0.1329	0.0501	14.13	26.4507
100	0.8754	14.5088	84.33	89.06	89.45	0.1316	0.0493	13.80	24.5997

## Data Availability

The dataset analyzed in this study is publicly available on Zenodo at https://doi.org/10.5281/zenodo.13885584 (accessed on 15 June 2026). This study used the Starlink-like subset of the synthetic LEO satellite trajectory dataset provided by Baudier et al. [22].
